# Safety and Feasibility of Long-term Intravenous Sodium Nitrite Infusion in Healthy Volunteers

**DOI:** 10.1371/journal.pone.0014504

**Published:** 2011-01-10

**Authors:** Ryszard M. Pluta, Edward H. Oldfield, Kamran D. Bakhtian, Ali Reza Fathi, René K. Smith, Hetty L. DeVroom, Masoud Nahavandi, Sukyung Woo, William D. Figg, Russell R. Lonser

**Affiliations:** 1 Surgical Neurology Branch, National Institute of Neurological Disorders and Stroke, National Institutes of Health, Bethesda, Maryland, United States of America; 2 Department of Neurological Surgery, University of Virginia Health Sciences Center, University of Virginia, Charlottesville, Virginia, United States of America; 3 Department of Neurosurgery, Kantonsspital Aarau AG, Aarau, Switzerland; 4 Molecular Pharmacology Section and Clinical Pharmacology Program, Medical Oncology Branch, Center for Cancer Research, National Cancer Institute, National Institutes of Health, Bethesda, Maryland, United States of America; Indiana University, United States of America

## Abstract

**Background:**

Infusion of sodium nitrite could provide sustained therapeutic concentrations of nitric oxide (NO) for the treatment of a variety of vascular disorders. The study was developed to determine the safety and feasibility of prolonged sodium nitrite infusion.

**Methodology:**

Healthy volunteers, aged 21 to 60 years old, were candidates for the study performed at the National Institutes of Health (NIH; protocol 05-N-0075) between July 2007 and August 2008. All subjects provided written consent to participate.

Twelve subjects (5 males, 7 females; mean age, 38.8±9.2 years (range, 21–56 years)) were intravenously infused with increasing doses of sodium nitrite for 48 hours (starting dose at 4.2 µg/kg/hr; maximal dose of 533.8 µg/kg/hr). Clinical, physiologic and laboratory data before, during and after infusion were analyzed.

**Findings:**

The maximal tolerated dose for intravenous infusion of sodium nitrite was 267 µg/kg/hr. Dose limiting toxicity occurred at 446 µg/kg/hr. Toxicity included a transient asymptomatic decrease of mean arterial blood pressure (more than 15 mmHg) and/or an asymptomatic increase of methemoglobin level above 5%. Nitrite, nitrate, S-nitrosothiols concentrations in plasma and whole blood increased in all subjects and returned to preinfusion baseline values within 12 hours after cessation of the infusion. The mean half-life of nitrite estimated at maximal tolerated dose was 45.3 minutes for plasma and 51.4 minutes for whole blood.

**Conclusion:**

Sodium nitrite can be safely infused intravenously at defined concentrations for prolonged intervals. These results should be valuable for developing studies to investigate new NO treatment paradigms for a variety of clinical disorders, including cerebral vasospasm after subarachnoid hemorrhage, and ischemia of the heart, liver, kidney and brain, as well as organ transplants, blood-brain barrier modulation and pulmonary hypertension.

**Clinical Trial Registration Information:**

http://www.clinicaltrials.gov; NCT00103025

## Introduction

Discovered by the late Robert J. Furchgott as an endothelium-derived relaxing factor [1], nitric oxide (NO) is a gas continuously synthesized and released from the vascular endothelium [1]. Preclinical studies indicate that NO may be beneficial for the treatment of experimental heart, liver, kidney and brain ischemia, as well as pulmonary hypertension [2], . Moreover, intra-arterially delivered NO has been shown to prevent the development of delayed cerebral vasospasm after subarachnoid hemorrhage [4], [5], [6], [7], [8], [9], [10] in a primate model and to facilitate treatment of brain tumors in a rodent model [11]. However, these preclinical findings have not been translated into effective clinical treatments, because safe and prolonged delivery of NO at therapeutic levels is not feasible using available systemic NO donors (nitroglycerin and nitroprusside) [4], [9] as these organic nitrates have unpredictable pharmacokinetics, rapidly develop tolerance, and when discontinued, often evoke rebound effect unless delivered extravascularly or locally [12]. Furthermore, it was believed that because of its chemical properties, NO in the presence of oxyhemoglobin is rapidly consumed and forms biologically inactive nitrate [13], [14], [15]. But, Mark Gladwin and his colleagues [16] showed that inhalation of NO produced a subtle increase in forearm blood flow associated with a concomitant increase in nitrite. As nitrite has forearm artery-to-vein gradients [17], these results suggest that nitrite is consumed across the vascular bed and is responsible for vasodilation earlier attributed exclusively to NO [16], [17]. Thus, an increasing body of evidence indicates that nitrite [16], [18] is a major intravascular storage pool for NO [17], [19], [20], [21] and can serve as an alternative to systemic delivery of NO or nitrates. Recently, intravenous administration of sodium nitrite was shown to prevent delayed cerebral vasospasm in a primate model of subarachnoid hemorrhage [22] and to limit ischemic damage to the heart, kidneys, liver and brain in animal models, doing so without a significant drop in blood pressure or other systemic toxicity [23], [24], [25].

Despite encouraging preclinical findings related to the use of sodium nitrite as an NO donor, clinical information regarding the prolonged intravenous use of nitrite is not available. To determine the safety, feasibility, and pharmacokinetics associated with a sodium nitrite infusion, we infused healthy volunteers with increasing doses of sodium nitrite for up to 48 hours. We hypothesized that continuous intravenous infusion of sodium nitrite is a safe method of NO delivery in doses that provide nitrite blood concentrations in a therapeutic range (1–25 µM) [17], [26], [27]. The goal of the study was to establish maximal tolerated dose, defined as the highest dose that does not cause side effects and dose limiting toxicity defined as the dose that evokes side effects particularly a drop of blood pressure and/or increase levels of methemogobin in blood. Details are described in the Materials and Methods. During infusion, we serially sampled blood for laboratory analysis to elucidate the NO metabolome.

## Results

### Subject characteristics

Twelve healthy subjects (5 males, 7 females) were included in this study. Their mean age was 38.8±9.2 years (range, 21–56 years). There were 2 Caucasians and 10 African Americans; this did not reflect a normal population distribution, it was the effect of the consecutive subject recruitment. Their mean weight was 77.8±19 kg (range, 49–115 kg). Preinfusion (baseline) demographics, sodium nitrite doses, methemoglobin, and NO metabolome data of the study group are presented in Tables 1 and 2.

**Table 1:** Baseline characteristics of healthy subjects.

Subject Number	Age	Sex	Race	Weight Kg	Dose mg/kg/hr	MABP mmHg	MetHb %
1	31	M	B	98	4.2	95	1
2	56	F	B	59	8.3	101	0.7
3	45	F	B	86	16.7	95	0.7
4	50	M	B	95	33.4	77	0.9
5	42	M	B	60	66.8	101	0.7
6	40	F	W	49	133.4	71	0.5
7*	34	F	B	69	266.9	100	0.7
8 †	47	M	W	65	533.8	85	0.5
9*	31	F	B	83	266.9	69	0.8
10*	33	M	B	87	266.9	85	0.6
11†	30	F	B	115	445.7	100	0.9
12†	27	F	B	68	445.7	86	0.8
Mean	39	F∶M	W∶B	77.8		89	0.7
SD	9	7∶5	2∶10	19		12	0.2

**Table 2:** Baseline characteristics of healthy subjects: nitric oxide (NO) metabolome.

Subject Number	Plasma NO_2_ µmol/L	Plasma NO_3_ µmol/L	Plasma SNO nmol/L	WholeBlood NO_2_ µmol/L	Whole Blood NO_3_ µmol/L	RBC NO_2_ µmol/L	RBC NO_3_ µmol/L
1	0.15	11.1	49.6	0.06	28.27		
2	0.07	13.28	35.2	0.23	24.65		
3	0.13	11.58	35.2	0.34	14.82		
4	0.24	15.41	22.8	0.22	15.23		
5	0.13	42.34	28.8	0.41	58.51		
6	0.14	43.63	46.3	0.42	62.99		
7*	0.11	14.23	30.11	0.28	14.11	0.2	14.11
8 †	0.33	27.22	18.93	0.24	27.37	0.3	23.84
9*	0.52	15.6	17.85	0.35	15.32	0.48	14.19
10*	0.16	12.29	22.77	0.16	40.65	0.39	20.26
11†	0.93	11.78	28.88	0.03	20.15	0.49	12.32
12†	0.13	9.13	15.8	0.15	10.26	0.29	8.61
Mean	0.25	18.97	29.35	0.24	27.69	0.36	15.56
SD	0.25	12.1	10.78	0.13	17.56	0.12	5.54

*maximal tolerated dose (MTD).

† dose limiting toxicity (DLT).

MABP, mean arterial blood pressure.

MetHb, methemoglobin.

RBC, red blood cells.

NO_2_, nitrite.

NO_3_, nitrate.

SNO, S-nitrosothiols.

SD, standard deviation.

### Sodium nitrite infusion and NO metabolome

We present the results in 3 groups based on the dosing scheme of sodium nitrite.

*Accelerated dosing* group (subjects 1–8; subjects 7 and 8 are presented separately in the maximal tolerated dose and dose limiting toxicity sections; respectively). The first subject underwent sodium nitrite infusion at 4.2 µg/kg/hr. Each subsequent subject received a sodium nitrite concentration double that of the preceding subject (details described in the Materials and Methods). Mean arterial blood pressure (Table S1) in this group of subjects was unaffected, and there was no evidence of toxicity during infusion until reaching subject 8 (dose, 533.8 µg/kg/hr; see the dose limiting dose section for details). Thirty minutes after the initiation of infusion, methemoglobin levels plateaued at 0.9% to 1.3% in subjects 1 through 6 and at 1.8% and 3.1% in subjects 7 and 8; respectively. Nitrite concentrations in plasma, whole blood and red blood cells increased in a dose- and time-dependent manner (Table S1). These levels reached a plateau between 20 minutes and 2 hours in all subjects. There was no clear dose- or time-dependent effect of the sodium nitrite infusion on nitrate concentrations in plasma or whole blood. However, nitrate concentrations in red blood cells increased in a dose- and time-dependent manner before reaching a plateau 12 hours after start of the infusion and returned to preinfusion levels 12 hours after the infusion ended. Plasma S-nitrosothiol concentrations reached a plateau at 6 hours after start of the infusion and returned to baseline within 12 hours after infusion cessation (Table S1).

### Maximal tolerated dose group (subjects 7, 9, and 10)

**Figure 1:**
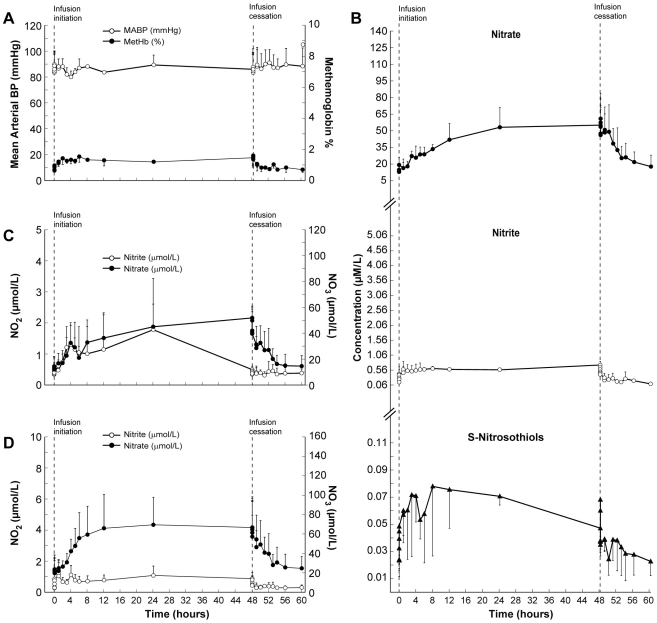
Clinical and biochemical data for the Maximal Tolerated Dose (MTD) group. This composite graph depicts the mean values and standard deviations (bars) of: A; mean arterial blood pressure and methemoglobin. B; NO metabolome concentrations in plasma (nitrite, nitrate, S-nitrosothiols), red blood cells (C; nitrite and nitrate) and whole blood (D; nitrite and nitrate) in three subjects who did not develop nitrite-related toxicity during sodium nitrite infusion at 266.9 µg/kg/hr [maximal tolerated dose (MTD)] Note that in this group all NO metabolome concentrations returned to baseline levels within 12 hours after completion of sodium nitrite infusion.

Subject 7 from the accelerated dosing group and the two additional volunteers (subjects 9 and 10) were infused at 266.9 µg/kg/hr. Sodium nitrite infusion did not affect mean arterial blood pressure in any of these subjects (Figure 1A). Methemoglobin levels increased gradually, reached a plateau 2 hours after beginning of the infusion (Figure 1A; maximal level, 1.6±0.5%), and remained elevated until 30 minutes after the infusion ended, before returning to baseline within 1 hour of infusion cessation.

Plasma nitrite concentrations increased, reaching a plateau 1.5 hours after infusion initiation (Figure 1B). Nitrite concentrations in red blood cells reached a maximum level 24 hours after beginning of the infusion (Figure 1C). Nitrite concentrations in whole blood increased from baseline before reaching a plateau 1 hour after infusion initiation (Figure 1D). Nitrite concentrations in all 3 compartments (plasma, red blood cells and whole blood) returned to baseline concentrations within 1 hour of infusion cessation.

Nitrate concentrations in plasma increased 12 hours after infusion initiation (Figure 1B), and reached their highest concentration 1 hour after infusion cessation. Nitrate concentrations in red blood cells increased 12 hours after infusion initiation, reaching the highest concentration immediately before cessation of the sodium nitrite infusion (Figure 1C). Nitrate concentrations in whole blood increased 8 hours after infusion initiation before reaching their highest-level 24 hours after infusion initiation (Figure 1D). Nitrate concentrations in the 3 compartments returned to baseline within 8 hours after infusion cessation (Figure 1B–D).

Concentrations of S-nitrosothiols in plasma reached their highest level at 8 hours after infusion initiation and returned to baseline within 5 minutes of infusion cessation (Figure 1B).

### Dose limiting toxicity group (subjects 8, 11, and 12)

This group included subject 8 (dose, 533.8 µg/kg/hr; Tables 1, 2) from the accelerated dosing group and two additional subjects that were infused with a dose of 445.7 µg/kg/hr (66% above the maximal tolerated dose of 266.9 µg/kg/hr). In this group, mean arterial blood pressure was decreased by more than 15 mmHg in two subjects, and the methemoglobin level exceeded 5% in one subject.

**Figure 2:**
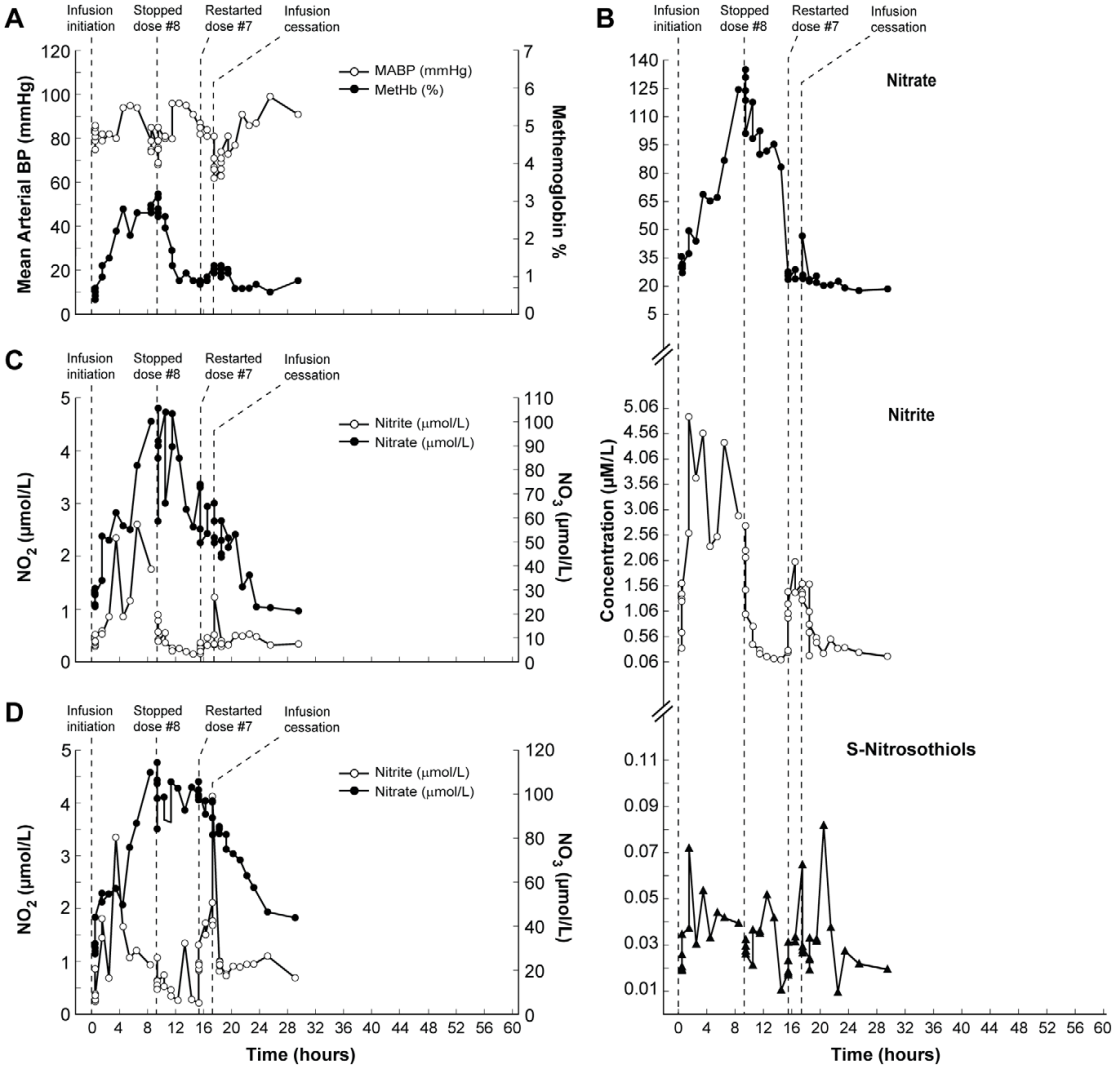
Clinical and biochemical data for Subject 8 (Dose Limiting Toxicity; DLT). This composite graph depicts: A; mean arterial blood pressure and methemoglobin; and NO metabolome concentrations in plasma (B; nitrite, nitrate, S-nitrosothiols), red blood cells (C; nitrite and nitrate) and whole blood (D; nitrite and nitrate) in subject 8 who had a drop of mean arterial blood pressure during sodium nitrite infusion at 533.8 µg/kg/hr and then again with the half-dose infusion (266.9 µg/kg/hr; MTD) despite 6 hours of rest between the infusions. The baseline values of mean arterial blood pressure and methemoglobin levels in this subject were 93 mmHg and 0.7%, respectively. He started sodium nitrite infusion at 533.8 µg/kg/hr. After 8 hours of sodium nitrite infusion, mean arterial blood pressure decreased by 15 mmHg and the infusion was stopped at the 9^th^ hour. After 6 hours of rest, the infusion was re-started at the half dose (266.9 µg/kg/hr). At this time blood pressure and methemoglobin levels, as well as nitrite and nitrate concentrations in plasma all returned to baseline levels. But nitrite in red blood cells and S-nitrosothiols in plasma as well as nitrate in red blood cells and whole blood remained elevated (B–D). Immediately after initiation of the second infusion, nitrite concentration in plasma increased rapidly. However, nitrate concentrations in plasma, red blood cells, and whole blood remained stable. At 2.25 hours the half-dose infusion was stopped because of a significant (this time more than 20 mmHg) decrease in blood pressure; methemoglobin barely increased at that time and nitrite and nitrate concentrations, despite being 2 and 8 times higher than at the beginning of the second infusion, remained well below the highest level during the first infusion (A–D). The subject remained asymptomatic during and after both infusions. All concentrations, except nitrite and nitrate in whole blood, returned to normal levels within 12 hours after stopping the infusion.

Nine hours after infusion initiation in subject 8, the mean arterial blood pressure dropped by 15 mmHg, and the infusion was stopped (Figure 2A). The methemoglobin level peaked at 3.1%. Six hours after interrupting the infusion, the infusion was re-started at half the starting dose (dose, 266.9 µg/kg/hr; Figure 2A). At that time, nitrite in red blood cells and S-nitrosothiols in plasma, as well as nitrate in whole blood and red blood cells, remained elevated. After re-initiation of the infusion, plasma nitrite concentrations increased rapidly (Figure 2B). At 2.25 hours, the infusion was stopped because the subject experienced a 20-mmHg decrease in mean arterial blood pressure (Figure 2A). All values except nitrite and nitrate in whole blood returned to baseline concentrations within 12 hours after infusion cessation (Figure 2A–D).

**Figure 3:**
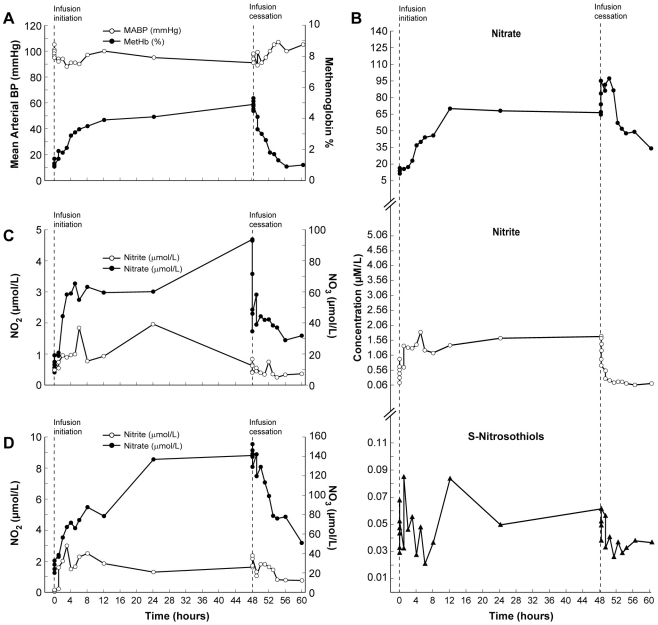
Clinical and biochemical data for Subject 11 (Dose Limiting Toxicity; DLT). This composite graph depicts: A; mean arterial blood pressure and methemoglobin and NO metabolome concentrations in plasma (B; nitrite, nitrate, S-nitrosothiols), red blood cells (C; nitrite and nitrate) and whole blood (D; nitrite and nitrate) in subject 11 who developed significant methemoglobinemia during sodium nitrite infusion at 445.7 µg/kg/hr. Mean arterial blood pressure remained stable during the infusion. The infusion remained uneventful until 2 and 5 minutes after the infusion cessation when methemoglobin level increased above 5% (A). However, it decreased during the next 5 minutes and returned to baseline at 8 hours after stopping sodium nitrite infusion. The subject remained asymptomatic. Within the NO metabolome only nitrite concentrations in plasma and red blood cells returned to baseline values; plasma nitrate, whole blood nitrite and nitrate, and S-nitrosothiols concentrations in plasma remained higher than the initial values until the end of the observation interval.

In subject 11, the methemoglobin level increased above 5% 2 minutes after sodium nitrite infusion cessation (Figure 3A,B). At the same time, nitrate concentrations in red blood cells and whole blood reached their highest levels (Figure 3C,D).

**Figure 4:**
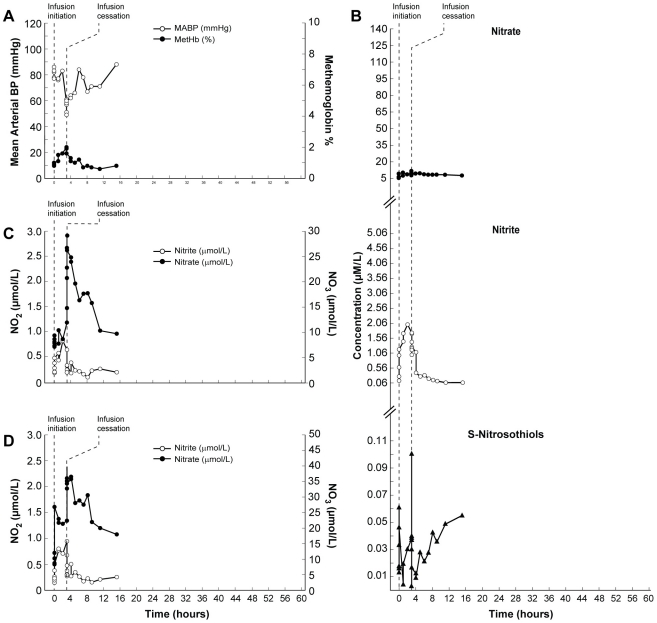
Clinical and biochemical data for Subject 12 (Dose Limiting Toxicity; DLT). This composite graph depicts: A; mean arterial blood pressure and methemoglobin and NO metabolome concentrations in plasma (B; nitrite, nitrate, S-nitrosothiols), red blood cells (C; nitrite and nitrate) and whole blood (D; nitrite and nitrate) in subject 12 who developed toxicity (significant drop of blood pressure) during sodium nitrite infusion at 445.7 µg/kg/hr. She was the second subject to have a sodium nitrite infusion at this dose. Three hours after initiation of sodium nitrite infusion, her mean arterial blood pressure decreased by 20 mmHg and the infusion was stopped. At this time her methemoglobin level was 2%, plasma level of nitrite was several times higher than baseline but nitrate remained unchanged. In the red blood cells, nitrite increased but nitrate remained at baseline level. Nitrite in whole blood was also several times higher than baseline and nitrate concentration was unchanged compared with baseline. S-nitrosothiol concentrations in plasma were at 37 nmol/L and well below the peak of 61 nmol/L reached a few hours earlier (A–D). This subject was observed for an additional 12 hours and remained asymptomatic. Among NO metabolome measurements in this subject, only S-nitrosothiols remained high 12 hours after stopping the infusion.

In subject 12, mean arterial blood pressure dropped by 20 mmHg 3.2 hours after infusion initiation, and the infusion was stopped. The methemogobin levels in this subject remained within normal values (Figure 4A). When the mean arterial blood pressure dropped in this subject, nitrite in plasma and nitrate in red blood cells and whole blood reached their highest levels (Figure 4B–D). In all three instances of dose limiting toxicity the subjects remained asymptomatic and the effects were transient and did not require additional intervention.

### Other clinical and laboratory analysis

None of the infused nitrite doses resulted in clinical symptoms. None of the patient complained of headache during or after sodium nitrite infusion. The initial mean arterial blood pressure for all 12 subjects was 87±10 mmHg, and 91±10 mmHg at 12 hours after stopping the sodium nitrite infusion (Table 3). Laboratory results remained unaffected by the infusion. Specifically, hematological, metabolic, and functional tests in blood did not show any changes in the kidney function (electrolytes, creatinine, uric acid, BUN) or liver functions (alanine transaminase, aspartate aminotransferase, direct bilirubin). Also, total protein, albumin, gamma-glutamyl transpeptidase, lactate dehydrogenase and total cholesterol were normal before, during and after the infusion as well as on follow up visit. The subnormal levels of urea nitrogen observed in several subjects before the infusion also remained low during and after the infusion.

**Table 3:** Characteristics of subjects at 12 hours after cessation of intravenous sodium nitrite infusion.

Subject Number	Dose µg/kg/hr	MABP mmHg	MetHb %	Plasma NO_2_ µmol/L	Plasma NO_3_ µmol/L	Plasma SNO nmol/L	Whole Blood NO_2_ µmol/L	Whole Blood NO_3_ µmol/L	RBC NO_2_ µmol/L	RBC NO_3_ µmol/L
1	4.2	101	0.5	0.11	6.99	31.21	0.18	19.29		
2	8.3	102	0.7	0.06	16.31	34.18	0.14	14.83		
3	16.7	88	0.8	0.11	15.25	26.79	0.11	15.05		
4	33.4	88	0.6	0.13	15.51	32.91	0.24	23.73		
5	66.8	102	0.6	0.24	12.34	28.2	0.39	18.24		
6	133.4	80	0.4	0.2	22.78	24.12	0.33	51.09		
7*	266.9	96	0.6	0.12	12.58	34.39	0.12	11.07	0.29	7.31
8†	533.8	91	0.9	0.17	18.52	19.42	0.69	43.79	0.34	21.21
9*	266.9	72	0.9	0.09	11.37	14.58	0.44	28.04	0.32	13.01
10*	266.9	97	0.6	0.11	29.64	18.88	0.33	34.52	0.54	23.4
11†	445.7	105	1	0.11	33.56	36.57	0.38	51.01	0.37	32.02
12†	445.7	88	0.8	0.01	7.68	54.98	0.26	18.02	0.20	9.68
Mean		93	0.7	0.12	16.88	29.69	0.3	27.4	0.34	17.77
SD		10	.2	0.06	8.18	10.59	0.17	14.36	0.11	9.42

7*The measurements were assessed at 12 hours after second (half-dose) infusion cessation.

*maximal tolerated dose (MTD).

† dose limiting toxicity (DLT).

MABP, mean arterial blood pressure.

MetHb, methemoglobin.

RBC, red blood cells.

NO_2_, nitrite.

NO_3_, nitrate.

SNO, S-nitrosothiols.

SD, standard deviation.

The *in vitro* mammalian chromosomal aberration test (Study No. AB39MP.341.BTL) was negative in all subjects. At 30 days follow-up, the subjects remained healthy, and their clinical and laboratory results were normal. There were no changes in the regularity of the menstrual cycle within 3 months after infusion.

### Nitrite pharmacokinetics

**Figure 5:**
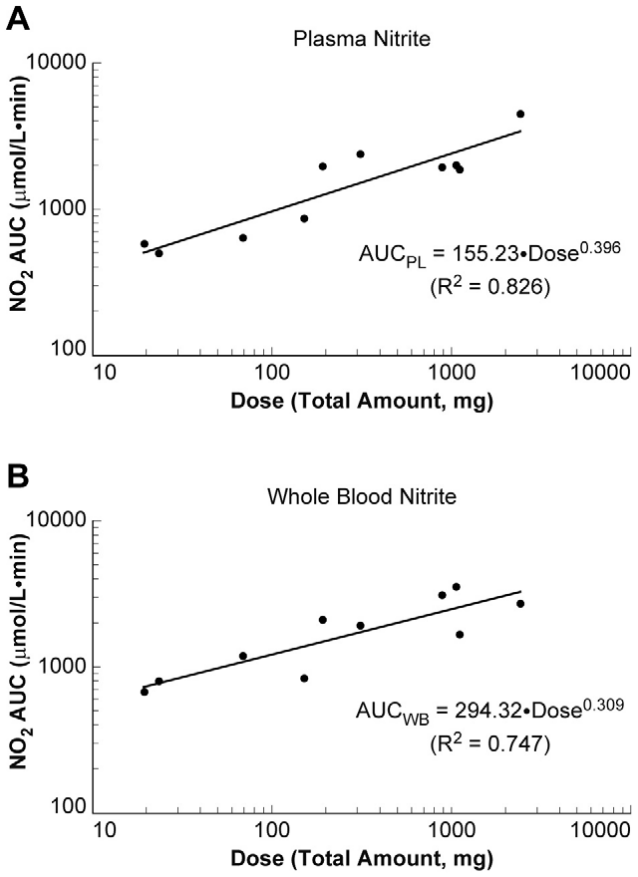
Nitrite pharmacokinetics in plasma and whole blood. Dose proportionality of nitrite in plasma (*left panel*) and in whole blood (*right panel*) was assessed using the area under the concentration-time curves AUC_0-last_ as a function of dose in milligrams. The estimated power coefficient (β_1_) for plasma and whole blood nitrite was significantly less than 1, indicating nonlinear pharmacokinetics of nitrite. This nonlinearity could be attributed to the increased nitrite clearance at the higher dose compared with the lowest dose, which strongly suggests that nitrite is much more rapidly removed from the body at higher doses.

Comparing nitrite the area under the concentration-time curves (AUC) relative to its corresponding dose (AUC_0-last_/D) over the entire dose range (Table 1) suggested that the systemic exposure to nitrite in plasma and whole blood increased less than proportionally with increasing doses. The estimated power coefficient (β_1_) of 0.396 (95% confidence intervals (CIs), 0.248–0.544) for plasma nitrite and 0.309 (95% CIs, 0.162–0.456) for whole blood nitrite (Figure 5A,B), which was significantly less than 1, clearly indicated nonlinear pharmacokinetics of nitrite. The observed nonlinearity appeared to be attributed to a dramatic increase in nitrite clearance up to 16-fold at 12.8 mg/kg or higher, compared with 7.5 mL/min/kg at the lowest dose of 0.2 mg/kg in the study, suggesting that nitrite is much more rapidly removed from the body at higher doses. The volume of distribution at steady state also appeared to increase at higher doses, but the values were highly variable across doses, and the trend was not as apparent as seen in clearance. The mean half-life of nitrite estimated at the maximal tolerated dose was 45.3±1.0 minutes for plasma and 51.4±22.6 minutes for whole blood.

### Chemiluminescence and gas chromatography-mass spectroscopy (GC-MS)

To compare the clinical usefulness and reliability of chemiluminescence and GC-MS for measurement of the NO metabolome, the nitrite concentrations were assessed in plasma and whole blood from subjects 9, 10, 11 and 12 using both methods. The baseline levels of nitrite in plasma and whole blood as measured by chemiluminescence (Table 2; 0.25±0.25 µmol/L, 0.24±0.13 µmol/L; respectively) were below the sensitivity of GC-MS (lower level of quantification [LLOQ] being 0.5 µmol/L). The mean values of nitrite in plasma during the infusion were 0.65±0.67 µmol/L by chemiluminescence method and 2.41±1.99 µmol/L by GC-MS. The plasma nitrite levels measured by both methods changed in parallel in a time- and dose-dependent manner. The mean values of nitrite concentrations in whole blood were also different when measured by chemiluminescence (0.58±0.4 µmol/L) and GC-MS (2.36±2.05 µmol/L). But, only whole blood nitrite levels measured by chemiluminescence changed in a time- and dose-dependent manner.

## Discussion

We established the maximal tolerated dose (266.9 µg/kg/hr) and dose limited toxicity (445.7 µg/kg/hr) for long-term intravenous infusion of sodium nitrite. The mean half-life of nitrite was 45.3 minutes for plasma and 51.4 minutes for whole blood, and was similar to the previously reported value for plasma (42.1 minutes) [26], [27]. The highest level of nitrite in blood of 3 volunteers in the maximal tolerated group was 1.1 µM (range: 0.62 µM to 1.17 µM), which would be within the therapeutic range according to preclinical and early clinical studies (range assessed as 1 µM to 25 µM). [26], [27], [28]. The highest nitrite blood levels in the dose limiting toxicity group were 3.4 µM in subject 8, 1 µM-subject 11, and 1.5 µM in subject 12.

The dose limiting toxicity was restricted to an asymptomatic increase of methemoglobin and, clinically irrelevant, a transient decrease of blood pressure. Furthermore, all hematological, metabolic and functional tests in blood did not show any changes in the blood chemistry, kidney, and liver functions during and after the infusion as well as on follow up visit. The nitrite pharmacokinetics analysis revealed a dramatic increase of nitrite clearance with increased doses. These findings suggest that prolonged intravenous infusion of sodium nitrite at doses at and below the maximal tolerated dose is safe and should assure therapeutic levels of nitrite.

These results should be valuable for designing human studies to investigate the utility of sodium nitrite for clinical disorders, which include cerebral vasospasm after subarachnoid hemorrhage [22], heart and brain ischemia and reperfusion, blood-tumor barrier modulation, pulmonary hypertension and organ transplantation [3], [11], [23], [24], [26], [29], [30], [31].

### Nitrite as endogenous NO donor

Nitric oxide, also known as endothelium-derived relaxing factor, is a free-radical gas [1], which exerts biological effects via activation of soluble guanylyl cyclase, leading to increased production of cyclic guanylyl monophosphate (cGMP) or via cGMP-independent pathways [14],[32]. Because of its free radical properties and extremely high affinity to ferrous iron of heme moiety, NO also forms nitrate [13], nitrosothiols, nitrosamines, and iron-nitrosyl compounds, collectively called NO-adducts (NOx) [33], [34] or NO metabolome. Each compound of NO metabolome has a potential to be a source of NO-related biological activity [13], [15], [32], [34], [35]. For many years it was widely accepted that in the presence of erythrocytes, NO is rapidly consumed [14], [15], but a seminal report showed that inhalation of NO produced a subtle increase in forearm blood flow despite regional inhibition of NO synthase associated with a concomitant increase in both heme-bound NO [15] and nitrite [16]. As nitrite has forearm artery-to-vein gradients in near-micromolar concentrations [17], these results suggest that nitrite is consumed across the vascular bed and is responsible for vasodilation that has been earlier attributed exclusively to NO [16], [17]. These findings suggest that sodium nitrite infusions resulting in near-physiologic local intravenous nitrite concentrations (1 µmol/L) can provide a reservoir for intravascular NO, particularly during inhibition of NO synthase, low oxygen tension, and in the presence of deoxygenated hemoglobin [17], [19], [22], [35], [36] acting as nitrite reductase [37].

### Preclinical study of nitrite as a local, on-demand NO donor

Low concentrations of nitrite in cerebrospinal fluid during cerebral vasospasm after subarachnoid hemorrhage [38], [39], [40] suggest that decreased NO availability is associated with the development of cerebral vasospasm [41]. Reversal and prevention of vasospasm by NO gas solution and by NO donors support this hypothesis [4], [9]. Furthermore, intravenous infusion of sodium nitrite at 10^−6^ M opens the brain-tumor barrier similarly to NO donors [11]. Thus, nitrite is an “on-demand” NO donor with deoxyhemoglobin acting as a nitrite reductase, especially under hypoxic conditions [17], [19], [37]. Similar conditions (i.e., the presence of deoxyhemoglobin [42] and lower pH [43], [44]) exist in the subarachnoid space after subarachnoid hemorrhage. Therefore, we used a long-lasting intravenous infusion of sodium nitrite after subarachnoid hemorrhage and demonstrated that infusion of sodium nitrite for 14 days prevented development of vasospasm in a primate model of subarachnoid hemorrhage [22].

### Safety and feasibility of long-term intravenous sodium nitrite infusion in humans

As the next step, before investigating the treatment of patients at risk of developing vasospasm after aneurismal subarachnoid hemorrhage, we established in this study the safety and toxicity of prolonged intravenous infusion of sodium nitrite in healthy volunteers. Toxicity was limited to asymptomatic transient decreases of arterial blood pressure and also asymptomatic increases of methemoglobin levels above 5%. Nitric oxide metabolome values returned to baseline levels within 12 hours; the only exception was S-nitrosothiols of subjects receiving toxicity-evoking doses.

Additional encouraging support of the clinical safety of prolonged sodium nitrite intravenous infusion was that we did not observe either tolerance, characteristic for organic nitrates, or clinically significant rebound effect often reported after discontinuation of nitroglycerin or sodium nitroprusside and the plasma clearance of nitrite was increasing with the dose increase.

Nitrite concentrations increased significantly slower and did not reach values predicted by preclinical and earlier clinical trials [26], [27]. Moreover, decreased mean arterial pressure was observed at lower nitrite concentrations than previously reported [17], [21]. Our data did not provide reliable answer whether a decrease of mean arterial pressure during the infusion of high doses of sodium nitrite was evoked by the presence of nitrite or S-nitrosothiols. Also, our data do not provide an explanation for the unexpected increase of methemoglobin levels during infusion of a relatively low dose of sodium nitrite in one subject.

### NO metabolome measurement

We confirmed that the concentrations of nitrite/nitrate and S-nitrosothiols could be reliably measured by chemiluminescence. The baseline values of the NO metabolome were within the range of earlier reported nitrite/nitrate/S-nitrosothiols concentrations in healthy subjects [17], [26], [45], [46]. The ion trap GC-MS method [47] provided inconsistent nitrite concentrations in whole blood samples. Also, the GC-MS lower level of quantification at 0.5 µM was below the baseline levels in our study (Tables 1, 2, and S1) and that reported by others [28], [48].

In conclusion: sodium nitrite intravenous infusion, which can be performed safely for prolonged intervals, provides levels of NO that are considered therapeutic. These results should be valuable for developing studies to investigate new NO treatment paradigms for a variety of clinical disorders, including cerebral vasospasm after subarachnoid hemorrhage and ischemia of the heart, liver, kidney, and brain, as well as organ transplants, blood-tumor barrier modulation and pulmonary hypertension.

## Materials and Methods

### Ethics statement

The protocol has been approved by the Combined Neuroscience Institutional Review Board and all subjects provided written consent to participate. The protocol for this trial and supporting CONSORT checklist are available as supporting information; see Checklist S1 and Protocol S1.

### Subjects

Healthy volunteers, aged 21 to 60 years old, were candidates for the study performed at the National Institutes of Health (NIH; protocol 05-N-0075) between July 2007 and August 2008.

### Exclusion criteria

Volunteers with a history or evidence of present or past hypertension (blood pressure greater than 140/90 mmHg), hypercholesterolemia (LDL cholesterol greater than 130 mg/dL) or diabetes mellitus (fasting blood glucose greater than 126 mg/dL) were ineligible. Volunteers who had a history of smoking within 2 years of study entry, cardiovascular disease, peripheral vascular disease, coagulopathy, or any other disease predisposing them to vasculitis or Raynaud's phenomenon were excluded. Also, volunteers with red blood cell G6PD deficiency, or a history of reacting to a medication or other substance characterized by dyspnea and cyanosis was excluded. Furthermore, volunteers with a baseline blood methemoglobin level greater than 1% with a blood pressure less than 100/70 mmHg on the study day and subjects treated with nitrates (e.g., nitroglycerin) were excluded.

### Study procedures

All studies were performed in the Intensive Care Unit (ICU) of the Clinical Center at the NIH. At the time of admission, each subject's clinical condition was assessed by the ICU staff and a study investigator. A full panel of blood and clinical chemistry tests, including urea nitrogen, alkaline phosphatase, and liver panel as well as a pregnancy test, were performed. A radial arterial line was placed for continuous blood pressure measurements. A peripherally inserted central catheter was placed in one arm and an antecubital intravenous line was placed in the other arm under local anesthesia. Thirty minutes after placement of arterial and venous access lines, the first baseline measurements of the investigated parameters were performed.

### Subject monitoring

**Figure 6:**
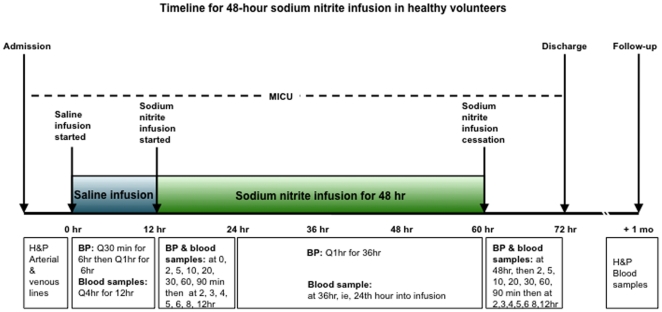
Time schedule of procedures and blood sampling for subjects participating in the safety, feasibility and pharmacokinetic study of long-term intravenous infusion of sodium nitrite. MICU; Medical Intensive Care Unit. H&P; history and physical. BP; arterial blood pressure. Mo; a month.

Because nitrite's half-life is short and onset of the expected effects is relatively brief (less than 30 minutes), the subject's general and neurological status was monitored and recorded every 1 to 2 hours for 12 hours after adjustment of a dose or termination of the infusion (Figure 6). During the entire study, mean arterial blood pressure, systolic and diastolic blood pressure, and EKG were continuously monitored. Subjects were also observed for other possible drug-related reactions. Blood methemoglobin was measured continuously using a Pulse CO-Oximeter RAD-57m (Masimo Corp., Irvine, CA).

Blood pressure, methemoglobin and blood samples to assess the NO metabolome, including nitrite and nitrate in plasma, whole blood and red blood cells, and the sum of protein and lipid bound NO (S-nitrosothiols) in plasma were collected directly before the infusion and then at 0, 2, 5, 10, 20, 30, 60, 90 minutes and 2, 3, 4, 5, 6, 8, 12, 24 and 48 hours (Figure 6) during sodium nitrite infusion. After the infusion was completed, blood samples were again collected at the same time intervals, with the last one collected at 12 hours after cessation of infusion.

During the follow-up visit, 30 days after the sodium nitrite infusion, blood samples for routine clinical chemistry and blood count were collected.

### Additional blood samples

Additional blood samples were collected just before starting sodium nitrite infusion to determine baseline parameters for platelet functions (Dr. J. Lozier, Clinical Laboratory Medicine Branch, Clinical Center, NIH), genotoxicity (chromosomal aberration studies; Bioreliance, Rockville, MD). To examine the influence of sodium nitrite on these measures, blood samples were collected again at 12 hours after cessation of sodium nitrite infusion. To assess the potential nitrite influence on the menstrual cycle, pre- and post-treatment menstrual cycle lengths were determined by subject reports.

### Sodium nitrite dosing

Sodium nitrite (Hope Medical Enterprises, Inc., Scottsdale, AZ) was formulated at a concentration of 300 mg in 10 mL water. A starting dose was 4.2 µg/kg/hr infused at 1 nmol/kg/min. It was predicted that this dose would result in a blood nitrite concentration of 77 nmol/L [27]. This concentration is well below expected normal levels (150 nmol/L) [27], [49]. Doses were increased on the basis of an accelerated titration scheme, in which the concentration of sodium nitrite was doubled for each subsequent subject until dose-limiting toxicity was reached. Following determination of dose limiting toxicity, 2 additional subjects were infused at the lower dose (half of the dose limiting toxicity dose) to expand the cohort to 3 subjects (the maximal tolerated dose group) and the next group of subjects had the dose increased by 66% (the dose limiting toxicity group) to define more precisely the toxic dose.

### Toxicity criteria

Toxicity related to sodium nitrite infusion was recognized when mean arterial blood pressure decreased more than 15 mmHg and lasted longer than 30 minutes or when symptomatic methemoglobinemia (>5%), shortness of breath, other breathing difficulties, or cardiovascular symptoms were observed. When toxicity occurred at a specific dose, the infusion was stopped for 6 hours, the dose was adjusted to half of the initial dose, and the subject continued in the study protocol. A blood pressure drop of greater than 20 mmHg for more than 30 minutes and/or methemoglobinemia above 5% and/or onset of clinical/EKG symptoms of cardio-respiratory problems required terminating the infusion.

### Blood sample preparation and assays for NO metabolome

Three milliliters of peripheral venous blood were drawn for each sample and immediately divided into 3 Eppendorf tubes, 2 containing 30 mL heparin (1000 U/mL) and 1 containing 250 µL stabilization solution (Ferricyanide, 12 mM; NEM, 10 mM; DTPA, 100 mM and 1% NP40 dissolved in nitrite-free Millipore water) to stabilize nitrite and S-nitrosothiols. The latter, a whole blood sample, was vortexed and stored immediately on dry ice. The heparinized blood was centrifuged at 2,500 rpm for 2 minutes (Galaxy Mini centrifuge; VWR International; West Chester, PA) at the bedside. Plasma was removed, aliquoted, frozen immediately on dry ice and stored at −80°C until assayed for NO metabolome. After removal of the buffy-coat, 100 µL of stabilization solution was added to each tube containing the red blood cell pellet of subjects 7 to 12. Each sample was re-vortexed, allowed to settle for 2 minutes and stored on dry ice, frozen and preserved. For the nitrite/nitrate measurements, the red blood cell pellet was removed and washed twice in 20 volumes of phosphate-buffered saline. Assays were performed in the Surgical Neurology Branch, National Institute of Neurological Disorders and Stroke at the NIH (chemiluminescence method, Sievers model 280i NO-analyzer; Boulder, CO) and by a commercial laboratory (Mass-spectroscopy method; Biopharmaceutical Research Inc.; Vancouver, BC, Canada).

### Ozone-based chemiluminescent assays

A stock solution of I_3_-NO reduction solution was prepared daily and 7 mL was poured into a glass purge vessel. Helium gas was bubbled under a constant pressure through the purge vessel, through 15 mL of 1M NaOH and then into the chemiluminescent NO analyzer (Sievers Model 280i NO analyzer, Boulder, CO), which can detect upwards of 1 pmol of NO gas. The following chemicals were placed in the purge vessel: 7 mL of glacial acetic acid, 2 mL of distilled water mixed with 111 mg of KI with addition of 0.072 g iodine (to yield a concentration of about 20–30 mM) and 50 mL antifoaming agent. Serial injections of S-nitrosoglutathione and nitrite were used for 4 hours to document the stability of the I_3_-stock solution exposed to air. There was no decrease in NO release from nitrite and S-nitrosothiols during this time period. This allowed for changing the reagent before each injection of the hemoglobin solutions and removed variability produced by increasing protein concentrations and foaming. NO release from S-nitrosothiols was eliminated by treating the samples with mercuric chloride (HgCl_2_, 5 mM) for two minutes. To specifically identify nitrite versus S-nitrosothiol, samples were incubated for three minutes with and without (9∶1, v/v; 5 wt./vol.%) acidified sulfanilamide, vortexed and reacted in the I_3_-reductant. The difference of sample concentrations treated with acidified sulfanilamide plus HgCl_2_ and concentration of samples treated with acidified sulfanilamide alone represents the concentrations of S-nitrosothiols.

To reduce foaming during analysis of nitrite and nitrate levels in plasma, samples were treated with a 1∶1 volume of ice-cold methanol and centrifuged at 17,900×g and 5°C for 5 minutes [36]. Nitrate was measured by reduction in vanadium (III) at 90°C; the following chemicals were mixed: 1 M hydrochloric acid (12 mL), vanadium chloride (0.8 g), distilled water (88 mL) and antifoaming agent (1 mL).

### Gas chromatography-mass spectroscopy (GC-MS) method

For validation and to compare the methods, 4 subjects (doses 7 and 7.1) had nitrite concentrations measured using mass spectroscopy. Details of the method used by Biopharmaceutical Research Inc. (Vancouver, BC) are protected by a patent. But generally the GC-MS method uses pentafluorobenzyl bromide for a sole derivatization of the blood sample. ^15^N-labeled nitrite is an internal standard and after analytical processes, ^14^N and ^15^N nitrite anions are separated in the mass spectrometer allowing for measurement of nitrite concentration in the sample with sensitivity of 22 fmol [45]. Previous baseline plasma nitrite levels measured by the GC-MS method in healthy volunteers were 1.8 µM [50].

### Pharmacokinetic analysis

Individual nitrite concentration versus time profiles were analyzed using WinNonlin (version 5; Pharsight Corp., Mountain View, CA) to obtain pharmacokinetic parameters such as the area under the concentration-time curves up to the last sampling point (AUC_0-last_), dose-normalized AUC_0-last_ (AUC_0-last_/D), clearance (CL), volume of distribution at steady-state (Vss), and terminal half-life. Two subjects who experienced toxicity were excluded from the pharmacokinetic analysis. Nitrite concentrations in plasma and in whole blood were used in the analysis without the endogenous baseline subtraction. Dose proportionality of nitrite pharmacokinetics was assessed by a regression analysis using a power model (e.g., AUC = β0·Doseβ1), which required 95% confidence intervals (CIs) of the power coefficient (β1), including 1, to demonstrate proportionality of the system (i.e., linear pharmacokinetics) [51].

### Statistical analysis

The strategy of this study was to establish the toxicity profile of prolonged intravenous infusion of sodium nitrite in healthy subjects. To achieve this goal, we analyzed blood pressure changes and blood nitrite, nitrate, S-nitrosothiols and methemoglobin levels of each subject before the infusion, during and after the infusion to determine the safe dose for future trials. Data are presented as mean value ± standard deviation (SD).

## Supporting Information

Table S1 NO metabolome data of subjects 1 through 6 from the dose acceleration group.
(0.41 MB DOC)

Checklist S1 CONSORT Checklist.
(0.03 MB DOC)

Protocol S1 Trial Protocol.
(0.10 MB DOC)
