# Supplementary material for: Safety and Feasibility of Long-term Intravenous Sodium Nitrite Infusion in Healthy Volunteers
Source: PLoS One. 2011 Jan 10;6(1):e14504. doi: 10.1371/journal.pone.0014504 (PMC3018414; doi:10.1371/journal.pone.0014504)
Supplement: Table S1 — NO metabolome data of subjects 1 through 6 from the dose acceleration group. (0.41 MB DOC) [file pone.0014504.s001.doc]

Table S1. NO metabolome data of subjects 1 through 6 from the dose acceleration group

| **Subject 1 Dosage** | **Time Point** | **MABP mmHg** | **MetHb %** | **Plasma NO_2_ µmol/L** | **Plasma NO_3_ µmol/L** | **Plasma SNO nmol/L** | **Whole Blood NO_2_ µmol/L** | **Whole Blood NO_3_ µmol/L** | **RBC NO_2_ µmol/L** | **RBC NO_3_ µmol/L** |
| --- | --- | --- | --- | --- | --- | --- | --- | --- | --- | --- |
| 4.2 µg/kg/hr | 0 Min | 95 | 0.5 | 0.15 | 11.09 | 73 | 0.06 | 28.27 |  |  |
| 4.2 µg/kg/hr | 2 Min | 99 | 1.1 | 0.20 | 12.74 | 60 | 0.08 | 19.17 |  |  |
| 4.2 µg/kg/hr | 5 Min | 91 | 1.1 | 0.16 | 9.54 | 107 | 0.06 | 24.51 |  |  |
| 4.2 µg/kg/hr | 10 Min | 96 | 1.2 | 0.13 | 9.98 | 102 | 0.06 | 21.19 |  |  |
| 4.2 µg/kg/hr | 20 Min | 100 | 1.1 | 0.29 | 8.79 | 138 | 0.09 | 23.15 |  |  |
| 4.2 µg/kg/hr | 30 Min | 96 | 1.0 | 0.25 | 9.54 | 47 | 0.14 | 19.11 |  |  |
| 4.2 µg/kg/hr | 60 Min | 105 | 0.8 | 0.27 | 9.47 | 82 | 0.13 | 21.67 |  |  |
| 4.2 µg/kg/hr | 90 Min | 109 | 0.5 | 0.25 | 9.63 | 68 | 0.12 | 16.19 |  |  |
| 4.2 µg/kg/hr | 2 Hr | 103 | 0.6 | 0.29 | 9.49 | 100 | 0.24 | 16.43 |  |  |
| 4.2 µg/kg/hr | 3 Hr | 105 | 1.0 | 0.20 | 9.53 | 39 | 0.18 | 14.48 |  |  |
| 4.2 µg/kg/hr | 4 Hr | 95 | 0.4 | 0.41 | 8.41 | 64 | 0.07 | 15.01 |  |  |
| 4.2 µg/kg/hr | 5 Hr | 99 | 0.4 | 0.32 | 8.59 | 82 | 0.19 | 17.57 |  |  |
| 4.2 µg/kg/hr | 6 Hr | 91 | 0.6 | 0.33 | 7.83 | 56 | 0.19 | 15.47 |  |  |
| 4.2 µg/kg/hr | 8 Hr | 103 | 0.6 | 0.34 | 9.19 | 51 | 0.27 | 13.09 |  |  |
| 4.2 µg/kg/hr | 12 Hr | 92 | 0.8 | 0.18 | 8.57 | 93 | 0.24 | 15.10 |  |  |
| 4.2 µg/kg/hr | 24 Hr | 91 | 0.6 | 0.13 | 8.96 | 48 | 0.22 | 14.55 |  |  |
| 4.2 µg/kg/hr | 48 Hr | 77 | 1.0 | 0.12 | 7.51 | 36 | 0.15 | 17.75 |  |  |
|  | 2 Min |  | 0.9 | 0.18 | 6.53 | 31 | 0.21 | 13.70 |  |  |
|  | 5 Min |  | 0.8 | 0.11 | 9.10 | 44 | 0.15 | 13.34 |  |  |
|  | 10 Min | 84 | 0.7 | 0.14 | 8.44 | 83 | 0.11 | 12.66 |  |  |
|  | 20 Min | 90 | 0.7 | 0.12 | 9.38 | 41 | 0.20 | 13.73 |  |  |
|  | 30 Min | 87 | 0.8 | 0.13 | 8.73 | 96 | 0.13 | 15.20 |  |  |
|  | 60 Min | 93 | 1.0 | 0.10 | 9.48 | 88 | 0.21 | 13.01 |  |  |
|  | 90 Min | 92 | 0.7 | 0.12 | 7.78 | 36 | 0.15 | 14.67 |  |  |
|  | 2 Hr | 108 | 0.6 | 0.13 | 10.45 | 56 | 0.16 | 13.71 |  |  |
|  | 3 Hr | 96 | 0.5 | 0.11 | 8.22 | 69 | 0.13 | 14.79 |  |  |
|  | 4 Hr | 97 | 0.5 | 0.12 | 10.75 | 62 | 0.15 | 13.99 |  |  |
|  | 5 Hr | 91 | 0.7 | 0.11 | 8.24 | 21 | 0.10 | 16.61 |  |  |
|  | 6 Hr | 89 | 0.6 | 0.12 | 8.11 | 33 | 0.18 | 12.91 |  |  |
|  | 8 Hr | 98 | 0.6 | 0.12 | 6.21 | 29 | 0.10 | 12.50 |  |  |
|  | 12 Hr | 101 | 0.5 | 0.11 | 7.00 | 31 | 0.18 | 19.29 |  |  |

| **Subject 2 Dosage** | **Time Point** | **MABP mmHg** | **MetHb %** | **Plasma NO_2_ µmol/L** | **Plasma NO_3_ µmol/L** | **Plasma SNO nmol/L** | **Whole Blood NO_2_ µmol/L** | **Whole Blood NO_3_ µmol/L** | **RBC NO_2_ µmol/L** | **RBC NO_3_ µmol/L** |
| --- | --- | --- | --- | --- | --- | --- | --- | --- | --- | --- |
| 8.3 µg/kg/hr | 0 Min | 89 | 0.6 | 0.07 | 13.28 | 35 | 0.23 | 24.65 |  |  |
| 8.3 µg/kg/hr | 2 Min | 94 | 0.5 | 0.09 | 10.56 | 35 | 0.20 | 20.30 |  |  |
| 8.3 µg/kg/hr | 5 Min |  | 0.6 | 0.07 | 11.04 | 42 | 0.18 | 25.60 |  |  |
| 8.3 µg/kg/hr | 10 Min | 96 | 0.6 | 0.14 | 12.56 | 49 | 0.17 | 19.81 |  |  |
| 8.3 µg/kg/hr | 20 Min | 93 | 0.5 | 0.16 | 12.09 | 50 | 0.18 | 25.23 |  |  |
| 8.3 µg/kg/hr | 30 Min | 92 | 0.4 | 0.14 | 14.75 | 43 | 0.20 | 29.37 |  |  |
| 8.3 µg/kg/hr | 60 Min | 87 |  | 0.11 | 12.89 | 49 | 0.20 | 27.41 |  |  |
| 8.3 µg/kg/hr | 90 Min | 78 | 0.7 | 0.16 | 10.97 | 43 | 0.19 | 34.01 |  |  |
| 8.3 µg/kg/hr | 2 Hr | 94 | 0.7 | 0.13 | 12.04 | 57 | 0.23 | 33.36 |  |  |
| 8.3 µg/kg/hr | 3 Hr | 76 | 0.7 | 0.22 | 14.22 | 77 | 0.25 | 39.87 |  |  |
| 8.3 µg/kg/hr | 4 Hr | 77 | 0.7 | 0.17 | 15.98 | 89 | 0.24 | 51.54 |  |  |
| 8.3 µg/kg/hr | 5 Hr | 72 | 0.9 | 0.13 | 12.33 | 76 | 0.26 | 27.23 |  |  |
| 8.3 µg/kg/hr | 6 Hr | 81 | 0.7 | 0.10 | 11.43 | 82 | 0.22 | 22.99 |  |  |
| 8.3 µg/kg/hr | 8 Hr | 100 | 0.7 | 0.13 | 10.05 | 70 | 0.19 | 23.16 |  |  |
| 8.3 µg/kg/hr | 12 Hr | 79 | 0.8 | 0.14 | 16.97 | 57 | 0.17 | 40.26 |  |  |
| 8.3 µg/kg/hr | 24 Hr | 72 | 0.8 | 0.18 | 11.22 | 50 | 0.30 | 36.36 |  |  |
| 8.3 µg/kg/hr | 48 Hr | 82 | 0.7 | 0.14 | 9.80 | 55 | 0.20 | 24.37 |  |  |
|  | 2 Min | 88 | 0.9 | 0.21 | 42.89 | 51 | 0.25 | 18.95 |  |  |
|  | 5 Min | 87 | 0.8 | 0.19 | 69.61 | 43 | 0.20 | 19.32 |  |  |
|  | 10 Min | 88 | 0.6 | 0.15 | 47.04 | 37 | 0.22 | 20.85 |  |  |
|  | 20 Min | 85 | 0.6 | 0.10 | 12.11 | 39 | 0.20 | 16.47 |  |  |
|  | 30 Min | 79 | 0.4 | 0.11 | 10.39 | 45 | 0.21 | 18.36 |  |  |
|  | 60 Min | 84 | 0.5 | 0.08 | 9.05 | 39 | 0.21 | 31.38 |  |  |
|  | 90 Min | 93 | 0.6 | 0.09 | 10.39 | 43 | 0.18 | 15.98 |  |  |
|  | 2 Hr | 78 | 0.8 | 0.10 | 12.97 | 53 | 0.18 | 16.40 |  |  |
|  | 3 Hr | 86 | 0.3 | 0.08 | 10.89 | 55 | 0.27 | 15.59 |  |  |
|  | 4 Hr | 73 | 0.8 | 0.07 | 10.42 | 45 | 0.18 | 9.05 |  |  |
|  | 5 Hr | 84 | 0.8 | 0.08 | 13.31 | 46 | 0.21 | 14.74 |  |  |
|  | 6 Hr | 79 | 0.5 | 0.09 | 11.16 | 42 | 0.20 | 15.69 |  |  |
|  | 8 Hr | 75 | 0.7 | 0.06 | 17.38 | 42 | 0.14 | 13.15 |  |  |
|  | 12 Hr | 89 | 0.7 | 0.06 | 16.30 | 34 | 0.14 | 14.83 |  |  |

| **Subject 3 Dosage** | **Time Point** | **MABP mmHg** | **MetHb %** | **Plasma NO_2_ µmol/L** | **Plasma NO_3_ µmol/L** | **Plasma SNO nmol/L** | **Whole Blood NO_2_ µmol/L** | **Whole Blood NO_3_ µmol/L** | **RBC NO_2_ µmol/L** | **RBC NO_3_ µmol/L** |
| --- | --- | --- | --- | --- | --- | --- | --- | --- | --- | --- |
| 16.7 µg/kg/hr | 0 Min | 95 | 0.7 | 0.13 | 11.58 | 35 | 0.34 | 14.83 |  |  |
| 16.7 µg/kg/hr | 2 Min | 96 | 0.8 | 0.11 | 12.82 | 36 | 0.48 | 17.39 |  |  |
| 16.7 µg/kg/hr | 5 Min |  | 0.7 | 0.13 | 13.88 | 32 | 0.59 | 14.05 |  |  |
| 16.7 µg/kg/hr | 10 Min | 100 | 0.7 | 0.12 | 13.29 | 48 | 0.72 | 15.78 |  |  |
| 16.7 µg/kg/hr | 20 Min | 96 | 0.8 | 0.13 | 11.37 | 56 | 0.54 | 16.18 |  |  |
| 16.7 µg/kg/hr | 30 Min | 92 | 0.9 | 0.11 | 11.50 | 63 | 0.57 | 14.49 |  |  |
| 16.7 µg/kg/hr | 60 Min | 94 | 0.9 | 0.12 | 11.23 | 66 | 0.56 | 17.88 |  |  |
| 16.7 µg/kg/hr | 90 Min | 93 | 0.6 | 0.11 | 12.74 | 69 | 0.51 | 13.92 |  |  |
| 16.7 µg/kg/hr | 2 Hr | 91 | 1.3 | 0.13 | 14.17 | 72 | 0.50 | 17.99 |  |  |
| 16.7 µg/kg/hr | 3 Hr | 84 | 0.8 | 0.18 | 12.59 | 119 | 0.45 | 13.99 |  |  |
| 16.7 µg/kg/hr | 4 Hr | 94 | 0.6 | 0.17 | 13.98 | 94 | 0.50 | 11.31 |  |  |
| 16.7 µg/kg/hr | 5 Hr | 84 | 0.6 | 0.22 | 13.78 | 52 | 0.33 | 11.35 |  |  |
| 16.7 µg/kg/hr | 6 Hr | 96 | 0.8 | 0.16 | 12.01 | 45 | 0.31 | 13.07 |  |  |
| 16.7 µg/kg/hr | 8 Hr | 97 | 0.4 | 0.15 | 11.02 | 43 | 0.32 | 36.23 |  |  |
| 16.7 µg/kg/hr | 12 Hr | 81 | 0.7 | 0.13 | 30.31 | 43 | 0.39 | 40.16 |  |  |
| 16.7 µg/kg/hr | 24 Hr | 77 | 0.7 | 0.12 | 29.77 | 34 | 0.33 | 22.75 |  |  |
| 16.7 µg/kg/hr | 48 Hr | 86 | 0.7 | 0.33 | 20.97 | 27 | 0.38 | 22.29 |  |  |
|  | 2 Min |  | 0.7 | 0.26 | 23.03 | 30 | 0.26 | 17.07 |  |  |
|  | 5 Min |  | 0.8 | 0.27 | 17.09 | 24 | 0.20 | 22.27 |  |  |
|  | 10 Min | 89 | 0.8 | 0.32 | 25.05 | 29 | 0.18 | 23.36 |  |  |
|  | 20 Min | 88 | 0.9 | 0.28 | 26.27 | 22 | 0.52 | 17.40 |  |  |
|  | 30 Min | 88 | 0.9 | 0.26 | 17.47 | 33 | 0.49 | 33.05 |  |  |
|  | 60 Min | 95 | 0.8 | 0.17 | 20.16 | 25 | 0.34 | 22.72 |  |  |
|  | 90 Min | 94 | 0.5 | 0.18 | 16.27 | 21 | 0.26 | 29.53 |  |  |
|  | 2 Hr | 96 | 0.8 | 0.20 | 14.24 | 29 | 0.22 | 18.81 |  |  |
|  | 3 Hr | 87 | 0.9 | 0.21 | 14.86 | 39 | 0.37 | 17.02 |  |  |
|  | 4 Hr | 94 | 0.7 | 0.19 | 16.76 | 39 | 0.16 | 5.43 |  |  |
|  | 5 Hr | 75 | 0.6 | 0.15 | 24.13 | 35 | 0.16 | 11.46 |  |  |
|  | 6 Hr | 77 | 0.9 | 0.12 | 24.56 | 28 | 0.12 | 15.72 |  |  |
|  | 8 Hr | 92 | 0.7 | 0.12 | 12.13 | 28 | 0.12 | 14.70 |  |  |
|  | 12 Hr | 88 | 0.8 | 0.11 | 15.25 | 27 | 0.11 | 15.05 |  |  |

| **Subject 4 Dosage** | **Time Point** | **MABP mmHg** | **MetHb %** | **Plasma NO_2_ µmol/L** | **Plasma NO_3_ µmol/L** | **Plasma SNO nmol/L** | **Whole Blood NO_2_ µmol/L** | **Whole Blood NO_3_ µmol/L** | **RBC NO_2_ µmol/L** | **RBC NO_3_ µmol/L** |
| --- | --- | --- | --- | --- | --- | --- | --- | --- | --- | --- |
| 33.4 µg/kg/hr | 0 Min | 77 | 0.9 | 0.24 | 15.41 | 23 | 0.22 | 15.23 |  |  |
| 33.4 µg/kg/hr | 2 Min |  | 0.9 | 0.22 | 14.67 | 18 | 0.30 | 14.56 |  |  |
| 33.4 µg/kg/hr | 5 Min |  | 0.9 | 0.20 | 10.36 | 38 | 0.30 | 17.72 |  |  |
| 33.4 µg/kg/hr | 10 Min | 82 | 0.9 | 0.27 | 13.48 | 45 | 0.29 | 15.49 |  |  |
| 33.4 µg/kg/hr | 20 Min | 80 | 0.9 | 0.23 | 14.07 | 42 | 0.39 | 18.11 |  |  |
| 33.4 µg/kg/hr | 30 Min | 77 | 0.9 | 0.32 | 15.17 | 41 | 0.34 | 14.62 |  |  |
| 33.4 µg/kg/hr | 60 Min | 77 | 0.8 | 0.25 | 13.30 | 35 | 0.27 | 13.54 |  |  |
| 33.4 µg/kg/hr | 90 Min | 82 | 0.8 | 0.27 | 12.76 | 45 | 0.35 | 12.84 |  |  |
| 33.4 µg/kg/hr | 2 Hr | 88 | 0.9 | 0.26 | 12.65 | 32 | 0.35 | 13.25 |  |  |
| 33.4 µg/kg/hr | 3 Hr | 78 | 0.9 | 0.24 | 15.84 | 53 | 0.45 | 14.07 |  |  |
| 33.4 µg/kg/hr | 4 Hr | 78 | 1.1 | 0.26 | 15.46 | 57 | 0.45 | 19.58 |  |  |
| 33.4 µg/kg/hr | 5 Hr | 76 | 0.7 | 0.24 | 15.31 | 68 | 0.42 | 16.31 |  |  |
| 33.4 µg/kg/hr | 6 Hr | 80 | 0.7 | 0.19 | 14.54 | 58 | 0.16 | 20.43 |  |  |
| 33.4 µg/kg/hr | 8 Hr | 77 | 0.7 | 0.19 | 11.61 | 61 | 0.32 | 17.18 |  |  |
| 33.4 µg/kg/hr | 12 Hr | 76 | 0.7 | 0.26 | 14.10 | 59 | 0.32 | 17.68 |  |  |
| 33.4 µg/kg/hr | 24 Hr | 76 | 0.7 | 0.28 | 19.60 | 38 | 0.18 | 19.99 |  |  |
| 33.4 µg/kg/hr | 48 Hr | 83 | 0.9 | 0.28 | 24.80 | 69 | 0.20 | 46.75 |  |  |
|  | 2 Min | 83 | 0.8 | 0.25 | 33.61 | 65 | 0.19 | 35.78 |  |  |
|  | 5 Min | 86 | 0.6 | 0.20 | 35.83 | 61 | 0.18 | 35.90 |  |  |
|  | 10 Min | 81 | 0.9 | 0.19 | 37.24 | 57 | 0.18 | 35.32 |  |  |
|  | 20 Min | 82 | 0.4 | 0.17 | 34.78 | 53 | 0.14 | 31.16 |  |  |
|  | 30 Min | 85 | 0.7 | 0.16 | 39.44 | 25 | 0.15 | 32.29 |  |  |
|  | 60 Min | 87 | 0.7 | 0.12 | 33.03 | 48 | 0.22 | 35.22 |  |  |
|  | 90 Min | 90 | 0.8 | 0.11 | 26.50 | 54 | 0.23 | 33.28 |  |  |
|  | 2 Hr | 94 | 0.8 | 0.09 | 25.51 | 48 | 0.24 | 46.06 |  |  |
|  | 3 Hr | 82 | 0.7 | 0.10 | 17.41 | 30 | 0.19 | 33.58 |  |  |
|  | 4 Hr | 84 | 0.7 | 0.08 | 14.93 | 29 | 0.22 | 25.76 |  |  |
|  | 5 Hr | 89 | 0.8 | 0.09 | 20.28 | 27 | 0.20 | 28.81 |  |  |
|  | 6 Hr | 83 | 0.7 | 0.13 | 20.84 | 30 | 0.20 | 25.68 |  |  |
|  | 8 Hr | 78 | 0.7 | 0.14 | 16.76 | 33 | 0.25 | 21.07 |  |  |
|  | 12 Hr | 88 | 0.6 | 0.13 | 15.51 | 33 | 0.24 | 23.73 |  |  |

| **Subject 5 Dosage** | **Time Point** | **MABP mmHg** | **MetHb %** | **Plasma NO_2_ µmol/L** | **Plasma NO_3_ µmol/L** | **Plasma SNO nmol/L** | **Whole Blood NO_2_ µmol/L** | **Whole Blood NO_3_ µmol/L** | **RBC NO_2_ µmol/L** | **RBC NO_3_ µmol/L** |
| --- | --- | --- | --- | --- | --- | --- | --- | --- | --- | --- |
| 66.8 µg/kg/hr | 0 Min | 101 | 0.7 | 0.13 | 42.34 | 29 | 0.41 | 58.51 |  |  |
| 66.8 µg/kg/hr | 2 Min |  | 0.6 | 0.14 | 50.58 | 32 | 0.55 | 62.67 |  |  |
| 66.8 µg/kg/hr | 5 Min |  | 0.5 | 0.30 | 50.27 | 38 | 0.57 | 54.27 |  |  |
| 66.8 µg/kg/hr | 10 Min | 101 | 0.7 | 0.28 | 42.68 | 22 | 0.58 | 47.07 |  |  |
| 66.8 µg/kg/hr | 20 Min | 105 | 0.8 | 0.35 | 48.61 | 39 | 0.44 | 59.68 |  |  |
| 66.8 µg/kg/hr | 30 Min | 105 | 0.8 | 0.50 | 46.78 | 31 | 0.37 | 62.79 |  |  |
| 66.8 µg/kg/hr | 60 Min | 105 | 0.8 | 0.45 | 44.20 | 20 | 0.67 | 63.71 |  |  |
| 66.8 µg/kg/hr | 90 Min | 100 | 0.6 | 0.60 | 49.40 | 29 | 0.48 | 53.61 |  |  |
| 66.8 µg/kg/hr | 2 Hr | 104 | 0.6 | 0.41 | 54.23 | 18 | 0.80 | 66.73 |  |  |
| 66.8 µg/kg/hr | 3 Hr | 90 | 1.0 | 0.72 | 34.47 | 34 | 0.56 | 50.39 |  |  |
| 66.8 µg/kg/hr | 4 Hr | 79 | 0.7 | 0.66 | 36.48 | 21 | 0.51 | 47.10 |  |  |
| 66.8 µg/kg/hr | 5 Hr | 87 | 0.8 | 0.80 | 32.58 | 18 | 0.81 | 29.01 |  |  |
| 66.8 µg/kg/hr | 6 Hr | 89 | 0.7 | 0.75 | 32.82 | 30 | 0.80 | 33.50 |  |  |
| 66.8 µg/kg/hr | 8 Hr | 99 | 0.8 | 1.29 | 46.82 | 23 | 0.87 | 45.52 |  |  |
| 66.8 µg/kg/hr | 12 Hr | 102 | 0.9 | 0.72 | 48.23 | 20 | 0.51 | 63.65 |  |  |
| 66.8 µg/kg/hr | 24 Hr | 97 | 0.7 | 0.63 | 36.22 | 34 | 0.61 | 48.12 |  |  |
| 66.8 µg/kg/hr | 48 Hr | 96 | 0.8 | 0.36 | 31.94 | 28 | 0.68 | 25.73 |  |  |
|  | 2 Min |  | 0.7 |  |  |  |  |  |  |  |
|  | 5 Min |  | 0.7 |  |  |  |  |  |  |  |
|  | 10 Min | 100 | 0.1 | 0.45 | 37.70 | 18 | 0.67 | 24.36 |  |  |
|  | 20 Min | 105 | 0.8 | 0.36 | 38.48 | 26 | 0.54 | 29.36 |  |  |
|  | 30 Min | 98 | 0.8 | 0.28 | 32.33 | 29 | 0.42 | 36.18 |  |  |
|  | 60 Min | 99 | 0.7 | 0.39 | 32.70 | 35 | 0.58 | 38.47 |  |  |
|  | 90 Min | 99 | 0.7 | 0.35 | 27.28 | 22 | 0.52 | 36.93 |  |  |
|  | 2 Hr | 99 | 0.5 | 0.24 | 22.47 | 29 | 0.36 | 33.70 |  |  |
|  | 3 Hr | 110 | 0.7 | 0.29 | 23.96 | 34 | 0.44 | 29.84 |  |  |
|  | 4 Hr | 98 | 0.9 | 0.30 | 18.67 | 30 | 0.44 | 30.06 |  |  |
|  | 5 Hr | 86 | 0.8 | 0.32 | 17.60 | 25 | 0.48 | 23.80 |  |  |
|  | 6 Hr | 87 | 0.6 | 0.25 | 16.40 | 24 | 0.38 | 23.00 |  |  |
|  | 8 Hr | 98 | 0.7 | 0.26 | 16.74 | 23 | 0.39 | 21.01 |  |  |
|  | 12 Hr | 102 | 0.6 | 0.24 | 12.34 | 28 | 0.36 | 18.24 |  |  |

| **Subject 6 Dosage** | **Time Point** | **MABP mmHg** | **MetHb %** | **Plasma NO_2_ µmol/L** | **Plasma NO_3_ µmol/L** | **Plasma SNO nmol/L** | **Whole Blood NO_2_ µmol/L** | **Whole Blood NO_3_ µmol/L** | **RBC NO_2_ µmol/L** | **RBC NO_3_ µmol/L** |
| --- | --- | --- | --- | --- | --- | --- | --- | --- | --- | --- |
| 133.4 µg/kg/hr | 0 Min | 71 | 0.5 | 0.14 | 43.63 | 46 | 0.42 | 62.98 |  |  |
| 133.4 µg/kg/hr | 2 Min | 72 | 0.6 | 0.15 | 43.89 | 52 | 0.50 | 75.05 |  |  |
| 133.4 µg/kg/hr | 5 Min | 69 | 0.9 | 0.33 | 43.23 | 54 | 0.52 | 63.31 |  |  |
| 133.4 µg/kg/hr | 10 Min | 69 | 1.0 | 0.31 | 41.46 | 38 | 0.53 | 58.12 |  |  |
| 133.4 µg/kg/hr | 20 Min | 69 | 0.7 | 0.39 | 40.17 | 24 | 0.40 | 57.51 |  |  |
| 133.4 µg/kg/hr | 30 Min | 63 | 0.6 | 0.55 | 38.81 | 43 | 0.34 | 53.60 |  |  |
| 133.4 µg/kg/hr | 60 Min | 67 | 0.5 | 0.50 | 38.86 | 31 | 0.61 | 50.33 |  |  |
| 133.4 µg/kg/hr | 90 Min | 65 | 0.6 | 0.66 | 37.97 | 22 | 0.44 | 57.09 |  |  |
| 133.4 µg/kg/hr | 2 Hr | 65 | 0.6 | 0.45 | 39.60 | 39 | 0.73 | 64.32 |  |  |
| 133.4 µg/kg/hr | 3 Hr | 72 | 0.6 | 0.80 | 39.39 | 24 | 0.51 | 61.89 |  |  |
| 133.4 µg/kg/hr | 4 Hr | 69 | 0.7 | 0.73 | 37.78 | 25 | 0.47 | 73.19 |  |  |
| 133.4 µg/kg/hr | 5 Hr | 76 | 0.2 | 0.88 | 33.38 | 21 | 0.74 | 76.94 |  |  |
| 133.4 µg/kg/hr | 6 Hr | 69 | 0.6 | 0.83 | 36.86 | 19 | 0.73 | 68.84 |  |  |
| 133.4 µg/kg/hr | 8 Hr | 74 | 0.5 | 1.43 | 35.98 | 45 | 0.80 | 58.83 |  |  |
| 133.4 µg/kg/hr | 12 Hr | 64 | 0.8 | 0.79 | 33.21 | 42 | 0.46 | 57.35 |  |  |
| 133.4 µg/kg/hr | 24 Hr | 66 | 0.5 | 0.75 | 28.80 | 35 | 0.56 | 49.27 |  |  |
| 133.4 µg/kg/hr | 48 Hr | 71 | 0.8 | 0.57 | 36.23 | 42 | 0.62 | 56.57 |  |  |
|  | 2 Min | 79 | 0.9 | 0.67 | 36.94 | 52 | 0.87 | 78.95 |  |  |
|  | 5 Min | 72 | 0.9 | 0.61 | 31.21 | 34 | 0.44 | 84.77 |  |  |
|  | 10 Min | 74 | 0.9 | 0.62 | 43.49 | 39 | 0.61 | 76.93 |  |  |
|  | 20 Min | 62 | 0.9 | 0.56 | 36.72 | 27 | 0.49 | 72.93 |  |  |
|  | 30 Min | 62 | 0.8 | 0.52 | 37.20 | 53 | 0.38 | 75.46 |  |  |
|  | 60 Min | 63 | 0.7 | 0.45 | 35.84 | 36 | 0.53 | 60.86 |  |  |
|  | 90 Min | 60 | 0.7 | 0.45 | 40.82 | 43 | 0.47 | 66.05 |  |  |
|  | 2 Hr | 64 | 0.5 | 0.31 | 32.75 | 19 | 0.33 | 69.19 |  |  |
|  | 3 Hr | 76 | 0.9 | 0.25 | 33.79 | 22 | 0.40 | 53.06 |  |  |
|  | 4 Hr | 66 | 0.7 | 0.33 | 26.28 | 27 | 0.40 | 50.21 |  |  |
|  | 5 Hr | 62 | 1.0 | 0.34 | 23.21 | 22 | 0.43 | 37.99 |  |  |
|  | 6 Hr | 75 | 0.4 | 0.28 | 22.59 | 19 | 0.35 | 40.81 |  |  |
|  | 8 Hr | 79 | 0.4 | 0.43 | 22.29 | 21 | 0.36 | 39.99 |  |  |
|  | 12 Hr | 80 | 0.4 | 0.20 | 22.78 | 24 | 0.33 | 51.09 |  |  |

MABP, mean arterial blood pressure.

MetHb, methemoglobin.

RBC, red blood cells.

NO_2_, nitrite.

NO_3_, nitrate.

SNO, S-nitrosothiols.
