# Supplementary material for: Safety and Feasibility of Long-term Intravenous Sodium Nitrite Infusion in Healthy Volunteers
Source: PLoS One. 2011 Jan 10;6(1):e14504. doi: 10.1371/journal.pone.0014504 (PMC3018414; doi:10.1371/journal.pone.0014504)
Supplement: Checklist S1 — CONSORT Checklist. (0.03 MB DOC) [file pone.0014504.s002.doc]

**CONSORT 2010 Flow Diagram**

**Allocation**

**Analysis**

**Follow-Up**

**Enrollment**

Assessed for eligibility (n= 25 )

Excluded (n=9)

♦  Not meeting inclusion criteria (n=2)

♦  Declined to participate (n= 6 )

♦  Other reasons (n= 1 )

Analysed (n= 12 )
♦ Excluded from analysis (give reasons) (n=0)

Lost to follow-up (give reasons) (n= 0)

Discontinued intervention (give reasons) (n=2) reached dose limiting toxicity

Allocated to intervention (n= 16 )

♦ Received allocated intervention (n= 12 )

♦ Did not receive allocated intervention (give reasons) (n= 4 ) 2-withdrawn for medical reasons, 2- declined to participate
