# Supplementary material for: Safety and Feasibility of Long-term Intravenous Sodium Nitrite Infusion in Healthy Volunteers
Source: PLoS One. 2011 Jan 10;6(1):e14504. doi: 10.1371/journal.pone.0014504 (PMC3018414; doi:10.1371/journal.pone.0014504)
Supplement: Protocol S1 — Trial Protocol. (0.10 MB DOC) [file pone.0014504.s003.doc]

**INTRODUCTION**

We invite you to take part in a research study at the National Institutes of Health (NIH).

First, we want you to know that:

Taking part in NIH research is entirely voluntary.

You may choose not to take part, or you may withdraw from the study at any time. In either case, you will not lose any benefits to which you are otherwise entitled. However, to receive care at the NIH, you must be taking part in a study or be under evaluation for study participation.

You may receive no benefit from taking part. The research may give us knowledge that may help people in the future.

Second, some people have personal, religious or ethical beliefs that may limit the kinds of medical or research treatments they would want to receive (such as blood transfusions). If you have such beliefs, please discuss them with your NIH doctors or research team before you agree to the study.

Now we will describe this research study. Before you decide to take part, please take as much time as you need to ask any questions and discuss this study with anyone at NIH, or with family, friends or your personal physician or other health professional.

Purpose of this study

The goal of this study is to examine the safety and effects of a new experimental drug called sodium nitrite in humans and to find the appropriate dose of sodium nitrite that may be used to prevent the tightening of the arteries (vasospasm) and to increase the blood flow in the human brain.

Facts that led us to this study

# Nitric Oxide (NO) has been used in animal studies to investigate human diseases, such as heart and brain ischemia or brain vasospasm, and to improve the ability of certain drugs to treat brain disorders. Recent studies have shown that (1) injections of sodium nitrite, which releases nitric oxide, improve blood flow and (2) injections over long periods of time prevent damage to the arteries in the brain. When damage to the arteries in the brain occurs, the brain’s blood vessels narrow. This narrowing of the blood vessels is called “Delayed Cerebral Vasospasm” and can cause stroke.

# Sodium nitrite has been approved by the FDA to treat cyanide poisoning and animal studies using nitrite have provided information regarding the side effects of long-term use of nitrite. However, despite research documenting the effects of such drugs and the safety of injections of nitrite in the blood, there is no information on safety and toxicity of intermediate (48 hours) and long-term (14 days) use of nitrite in humans. A 14-day nitrite infusion is proposed in this study because it is the time frame in which vasospasm develops after damage of blood vessels in the brain.

Therefore, we invite you to participate in this study as a healthy volunteer to help us find a safe treatment for vasospasm in humans and to possibly improve the condition of those who suffer from vasospasm.

Study population

~~A total of 9 healthy volunteers will be enrolled in this study.~~  **A total of 25 healthy volunteers will be enrolled in this study. The first group of 16 patients will have sodium nitrite infused intravenously in increasing concentrations for 48 hours. After this group has been studied, a second group of 9 patients will have continuous intravenous infusion of sodium nitrite for 14 days (three patients at each of three different doses).**

Who Can Be In This Study

To be eligible for this research study, you must

- be 21-60 years of age.
- be in good health.
- be willing to temporarily stop any medications (except oral contraception), vitamin supplements, herbal medicines.
- have a negative pregnancy test, if a female subject of childbearing age**, and agree to track your menstrual cycle for four months.**
- **have hemoglobin levels above 11.1 g/dL.**

Who May Not Be In This Study

You may not be eligible for this research study if

- you have a history or evidence of present high blood pressure >140/90 mmHg, hypercholesterolemia (LDL cholesterol >130 mg/dL), or diabetes mellitus (fasting blood glucose >126 mg/dL),
- you have a history of smoking within the past two years,
- you have a history of cardiovascular disease, peripheral vascular disease, coagulopathy, or any other disease predisposing to vasculitis or Raynaud's phenomenon.
- you have a red blood cell deficiency (G6PD),
- you have a history of allergies to medicines or other substances for dyspnea and cyanosis.
- you have an initial methemoglobin level > 1%,
- you are breast-feeding, since nitrite could cause methemoglobinemia in the infant,
- you have a blood pressure of less than 100/70 mmHg on the study day,
- you are pregnant, since nitrite may affect the fetus.
- **you take nitrate medications (i.e. nitroglycerin).**

Procedures

All studies will be performed in the Medical ICU unit of the Critical Care Medicine Department (CCMD) and the Neuroscience Nursing Unit of the Clinical Center at the NIH. You will be asked to refrain from drinking alcohol and beverages containing caffeine for at least 12 hours prior to the study. The infusion will last for **48 hours or 14 days, and you will stay in the hospital for a todal of three days (48-hour infusion) or 16 ¼ days (14-day infusion).**~~a total of 16 ¼ days, because this is the time frame in which vasospasm develops after the tearing of an enlarged blood vessel in the brain.~~

After signing the consent form, you will be admitted to the Medical ICU for **three days (48-hour infusion) or for** two days **(14-day infusion)**. At the time of admission, we will ask you about your medical history, and give you a cardiovascular exam, physical examination, a pregnancy test (if a female subject of childbearing age), and collect your blood for routine chemistry analyses, blood gas analysis, coagulation, liver function tests, and a red blood cell count.

We will insert a thin plastic tube (a catheter) into one of the arteries in your wrist or elbow crease area to measure your blood pressure. If the catheter is to be inserted into the wrist, a simple test will be conducted prior to insertion to ensure that both of the arteries in your wrist are working. A small amount of a local anesthetic (numbing medicine) will be used to numb the skin over the artery. Then, the catheter will be placed in the artery with a needle. The needle will be removed, leaving only the thin catheter in the artery. The catheter will be fastened to the skin with tape. We will also insert a thin plastic tube (catheter) into one arm vein in each of your arms. **During the time the catheters are in place, you should limit the movement of your arm to avoid moving the catheters. A trained staff member will be available at all times and should be notified immediately if you have pain or discomfort.** The catheters will be used to give you the nitrite and to draw blood from you. The catheters will be taped to the skin to hold it in place. Thirty minutes after admission, we will take your blood pressure, collect blood from you and monitor your heart rate.

After we take your measurements, we will start an infusion (continuous injection) of saline (0.9% NaCl) into the catheter in your arm. We will monitor your blood pressure and heart rate every thirty minutes for six hours, and then every hour for six hours, and then every two hours for twelve hours. We will collect 1ml of your blood (less than 1/5 of the teaspoon) every 4 hours for **12 hours (48-hour infusion) or** 24 hours **(14-day infusion)**. **During these 12 hours (48-hour infusion) and o**n the second day **(14-day infusion)**, we will start the infusion of sodium nitrite in 0.9% NaCl (saline) as a solvent. The amount of sodium nitrite received will **depend on which study group you are in, and also by** your body size. **In the 48-hour infusion study, the dose will be increased from 6 mg/day to 3,052 mg/day maximum. The dose in the 14-day infusion group will be determined from the maximum tolerated dose given in 48-hour infusion group.**  For example, **if the maximum tolerated dose in the 48-hour infusion group is 44 µmol/min, and you are one of the first three patients in the 14-day infusion group and you weigh 70 kg, you will**  ~~if you weigh 70 kg you will~~ receive sodium nitrite **infusion** at 11 µmol/min for 14 days. Your blood pressure and levels of methemoglobin (which temporarily and slightly lowers the oxygen carried in the red blood cells) will be monitored closely. We will monitor your blood pressure every 10 minutes for the first 2 hours. If the blood pressure remains stable, the frequency of measurements will decrease to every 30 minutes for 4 hours and then to every 1 hour for the next 6 hours, and then every 4 hours for 12 hours. If the blood pressure continues to remain stable, we will only monitor you every 8 hours for the rest of the study. We will draw your blood to determine how much nitrite and methemoglobin you have in your body several times during the first hour of the infusion, then every hour for several hours, and then at 8, 12 and 24 hours and, lastly, every 24 hours for the rest of the nitrite infusion (total of 14 days).

**If you are in the 48-hour infusion group, you will stay in the Medical ICU through the whole study (72 hours). Your total admission time will be 72 hours. During the last 12 hours of the study the blood pressure and blood samples will be collected as during the first 12 hours of sodium nitrite infusion.**

**If you are in the 14-day infusion group, a**fter the initial 24 to 48 hours of nitrite infusion you will be transferred to the nursing unit for the rest of the study. In the nursing unit, the ~~you will continue a~~ sodium nitrite infusion **will continue**. When the nitrite infusion is complete (15^th^ day of the study and 14^th^ day of nitrite), you will be observed for an additional 24 hours. Your blood pressure will be monitored and your blood will be collected frequently, at the same schedule as the first day of nitrite infusion. The total time of your admission will be 16 ¼ days.

You will have an additional **outpatient** check up at the NIH 30 days after the study because of the unknown effects of long-term use of sodium nitrite.

Risks, Inconveniences and Discomforts

#### **Sodium nitrite**

Sodium nitrite given through injection is currently available and approved by the FDA for use in the emergency treatment of cyanide poisoning. It has been used commercially as a food preservative, an anti-corrosive agent, a coloring agent, and an anti-heart attack agent, as well as a laxative, burn ointment, and liniment. Side effects of excess exposure to nitrite **include** nausea, vomiting, abdominal pain, dizziness, headache, flushing, cyanosis, fast breathing, shortness of breath, low blood pressure and death. You may appear to have skin discoloration and experience difficulty **breathing**, seizures, low blood pressure, coma and death if your methemoglobin level rises above your hemoglobin level. We will monitor your levels of methemoglobin, **blood pressure and breathing rate** closely and stop the nitrite infusion if **you develop any signs or symptoms of toxicity.** **~~the level increases~~**.

***Risks of nitrite infusion***

Short-term use of sodium nitrite has been tested before in healthy volunteers **and proven to be safe.** **Intermediate and** long-term use have not been tested and there may be unknown and unexpected side effects. Side effects of the use of nitrite in the blood include a change of red blood cells that carry oxygen in the blood to methemoglobin, which slightly and temporarily blocks oxygen being carried in the blood. Additional risks related to methemoglobinemia include shortness of breath and bluish discoloration of the lips and skin, platelet dysfunction including hemorrhage and nitric oxide release including a decrease in blood pressure. To decrease these risks of sodium nitrite infusions, the methemoglobin/nitrite level and blood pressure, as well as platelet functions, will be closely monitored. In past studies, the only side effects were a decrease of blood pressure for no more than 30 to 60 minutes after infusion and an increase of methemoglobin levels in blood. Low blood pressure and methemoglobinemia may lead to shortness of breath, light-headedness and even stroke. You will be examined often for any neurological symptoms and changes in blood pressure and blood **methemoglobin** levels. You will be monitored for 24 hours after the discontinuation of infusion of the drug and then again during the follow-up visit at the NIH four weeks after receiving the drug. If any adverse events develop between time of the nitrite infusion and the NIH follow-up visit, you may contact us to obtain professional advice and/or help.

***Other possible side effects of nitrite infusion:***

**If you are a woman:**

**A prolonged estrous cycle, which is equivalent to the menstrual cycle in humans, was observed in mice receiving average daily dosages of nitrite of 200 mg/kg or greater. Thus, to assess a potential nitrite influence on the menstrual cycle, we will ask you to track the first day of your menstrual cycle on a calendar for two months before and two months after receiving nitrite infusion. The calendar will be collected at study visits and you will be asked to call in to report the first day of your menstrual cycle.**

**If you are a man:**

**Toxicology data in rats and mice show that an average oral daily dose of more than 200 mg/kg for two years decreases sperm motility. This dose is many times higher than the dose you will receive in our studies. At lower dose levels in animals, sperm motility was not affected. There is no data in humans regarding the effect of intravenous nitrite administration of sperm motility. Furthermore, due to the high variability in semen parameters (sperm count, maturity, viability, and motility) depending on semen collection techniques, ejaculatory frequency, and period of abstinence before semen collection, we will not be able to assess accurately sperm quality and motility after the short-term sodium nitrite infusion used in the current study.**

## ***Risks of Arterial Radial Catheterization***

Some discomfort is associated with the placement of the arterial needle. Bruising or swelling at the site of the arterial catheter occurs in 5 to 20 percent of patients, but is only temporary. Fainting is remotely possible. Blockage of the artery by the catheter can occur, but is unlikely because the catheter is left in for only a short period of time. There have been two delayed complications from wrist arterial catheters at NIH. One person developed a small radial artery aneurysm 2 years after arterial catheterization. A second developed a blocked artery a few days after arterial catheterization. Both required surgery. A very remote risk for clotting and tearing of arteries and veins has been reported in literature; however, we have performed arterial catheterizations in more than 70 individuals without these complications.

## ***Risks of venous catheter (PICC)***

The risks of an intravenous catheter include local bleeding, infection, or local inflammation of the skin and vein with pain and swelling. The catheters are placed routinely by trained staff in the Medical ICU. Other complications may include incorrect positioning of the catheter (< 5%), damage to the artery (< 1%), and bruising. Very rare complications include spinal nerve injury, significant bleeding and positioning of the catheter outside of the vein. The risk of infection after 24 hours of catheter placement is minimal. However, the risk of infection significantly increases with time. If infection develops, the catheter will be removed, antibiotics will be started and new i.v. access will be established for continuation of the sodium nitrite infusion and antibiotic administration as requested by the Infectious Diseases service.

## ***Risks of blood tests***

A total of less than 300 ml (an 8-ounce glass) of blood will be collected in this study. During the study, blood will be collected from the catheters placed in your arms, so no additional needle sticks will be performed. We will draw your blood again approximately 4 weeks after the nitrite infusions to repeat blood chemistries for comparison. You may experience some discomfort at the site of the needle entry and there is a remote risk of infection.

Use of stored samples

Samples will be stored in secured freezers on the NIH campus. The samples will be inventoried by codes we assign. The key to the code will be kept in a separate and secure area. These samples will be used for the study described in this consent form.

Potential Benefits

There is no direct benefit to you; however, knowledge gained from this study may lead to new therapeutic options for patients with vasospasm, cardiovascular, hematologic, and respiratory diseases.

Right of Withdrawal and Conditions for Early Withdrawal

You may withdraw from the study at any time and for any reason without loss of benefits or privileges to which you are otherwise entitled. The investigator can remove you from the study at any time, if she or he believes that continuation is not in your best medical interest or if you are unable to comply with the requirements of the study.

Results from this study : Limited Access to Research Results

The investigators will not provide you with the results of any research tests or evaluations because the results are preliminary and further research may be necessary before they are fully understood*.* However, if information is developed from this study that may be important for your health, you will be informed when it becomes available.

By agreeing to participate in this study, you do not waive any rights that you may have regarding access to and disclosure of your records.

Alternatives to Participation or Treatment

This study is recruiting healthy volunteers. The alternative to participating in this study is to not participate.

Confidentiality

All medical information collected will be kept in a locked file at the Clinical Center at the NIH. Codes will be used to label all patient data and only the research team will have access to the code. Blood specimens will be coded to ensure patient confidentiality. All data will be maintained in computers with password protection. Strict standards of confidentiality will be upheld at all times.

Compensation

You will be compensated for research-related discomfort and inconvenience in accord with NIH guidelines. If you are unable to finish the study, you will be paid for those parts completed.

Group 1:

- Hours: 3 days x $40 = $120
- Inconvenience units (1 unit = $10)
  - i.v. catheter placement, 5 units = $50
  - intra-arterial catheter placement, 21 units = $210
  - blood sample, 32 units = $320
  - Total: 58 inconvenience units = $580
- Total for study: $120 + $580 = $700

Group 2:

- Hours: 16 ¼ days x $40 = $650
- Inconvenience units (1 unit = $10)
  - - - i.v. catheter placement 10 units = $100
      - intraarterial catheter placement, 21 units = $210
      - blood sample, 51 units = $510
      - Total: 82 inconvenience units = $820
- Total for study: $650 + $820 = $1470

**OTHER PERTINENT INFORMATION**

**1. Confidentiality.** When results of an NIH research study are reported in medical journals or at scientific meetings, the people who take part are not named and identified. In most cases, the NIH will not release any information about your research involvement without your written permission. However, if you sign a release of information form, for example, for an insurance company, the NIH will give the insurance company information from your medical record. This information might affect (either favorably or unfavorably) the willingness of the insurance company to sell you insurance.

The Federal Privacy Act protects the confidentiality of your NIH medical records. However, you should know that the Act allows release of some information from your medical record without your permission, for example, if it is required by the Food and Drug Administration (FDA), members of Congress, law enforcement officials, or other authorized people.

**2. Policy Regarding Research-Related Injuries.** The Clinical Center will provide short-term medical care for any injury resulting from your participation in research here. In general, no long-term medical care or financial compensation for research-related injuries will be provided by the National Institutes of Health, the Clinical Center, or the Federal Government. However, you have the right to pursue legal remedy if you believe that your injury justifies such action.

**3. Payments.** The amount of payment to research volunteers is guided by the National Institutes of Health policies. In general, patients are not paid for taking part in research studies at the National Institutes of Health.

**4. Problems or Questions.** If you have any problems or questions about this study, or about your rights as a research participant, or about any research-related injury, contact the Principal Investigator, **Edward H.Oldfield**; Building **10**, Room **5D37**, Telephone: **301-496-5728**. Other researchers you may call are: **Dr. Ryszard Pluta 301-594-8117; pager 104-5452; pager (NIH operator) 301-496-1211**

You may also call the Clinical Center Patient Representative at 301-496-2626.

**5. Consent Document.** Please keep a copy of this document in case you want to read it again.

| **COMPLETE APPROPRIATE ITEM(S) BELOW:** | |  | |
| --- | --- | --- | --- |
| **A. Adult Patient’s Consent** | **B. Parent’s Permission for Minor Patient.** |  | |
| I have read the explanation about this study and have been given the opportunity to discuss it and to ask questions. I hereby consent to take part in this study. | I have read the explanation about this study and have been given the opportunity to discuss it and to ask questions. I hereby give permission for my child to take part in this study.  (Attach NIH 2514-2, Minor’s Assent, if applicable.) |  | |
|  |  |  | |
| Signature of Adult Patient/Legal Representative Date | Signature of Parent(s)/Guardian Date |  | |
|  |  |  | |
| **C. Child’s Verbal Assent (If Applicable)**  The information in the above consent was described to my child and my child agrees to participate in the study. | |  |  |
|  |  |  | |
| Signature of Parent(s)/Guardian Date |  |  | |
|  |  |  | |
| **THIS CONSENT DOCUMENT HAS BEEN APPROVED FOR USE  FROM NOVEMBER 17, 2005 THROUGH NOVEMBER 17, 2006.** | |  |  |
|  |  |  | |
| Signature of Investigator Date | Signature of Witness Date |  | |
